# Aciculatin Induces p53-Dependent Apoptosis via MDM2 Depletion in Human Cancer Cells *In Vitro* and *In Vivo*

**DOI:** 10.1371/journal.pone.0042192

**Published:** 2012-08-13

**Authors:** Chin-Yu Lai, An-Chi Tsai, Mei-Chuan Chen, Li-Hsun Chang, Hui-Lung Sun, Ya-Ling Chang, Chien-Chih Chen, Che-Ming Teng, Shiow-Lin Pan

**Affiliations:** 1 Pharmacological Institute, College of Medicine, National Taiwan University, Taipei, Taiwan; 2 Institute of Biotechnology and Pharmaceutical Research, National Health Research Institutes, Zhunan Town, Taiwan; 3 Department of Biotechnology, Hungkuang University, Taichung, Taiwan; University of Sherbrooke, Canada

## Abstract

Aciculatin, a natural compound extracted from the medicinal herb *Chrysopogon aciculatus*, shows potent anti-cancer potency. This study is the first to prove that aciculatin induces cell death in human cancer cells and HCT116 mouse xenografts due to G1 arrest and subsequent apoptosis. The primary reason for cell cycle arrest and cell death was p53 accumulation followed by increased p21 level, dephosphorylation of Rb protein, PUMA expression, and induction of apoptotic signals such as cleavage of caspase-9, caspase-3, and PARP. We demonstrated that p53 allele-null (−/−) (p53-KO) HCT116 cells were more resistant to aciculatin than cells with wild-type p53 (+/+). The same result was achieved by knocking down p53 with siRNA in p53 wild-type cells, indicating that p53 plays a crucial role in aciculatin-induced apoptosis. Although DNA damage is the most common event leading to p53 activation, we found only weak evidence of DNA damage after aciculatin treatment. Interestingly, the aciculatin-induced downregulation of MDM2, an important negative regulator of p53, contributed to p53 accumulation. The anti-cancer activity and importance of p53 after aciculatin treatment were also confirmed in the HCT116 xenograft models. Collectively, these results indicate that aciculatin treatment induces cell cycle arrest and apoptosis via inhibition of MDM2 expression, thereby inducing p53 accumulation without significant DNA damage and genome toxicity.

## Introduction

Discovery and development of anti-cancer drugs is an endless challenge for biochemists and pharmacologists worldwide. Natural products are abundant sources of novel and cytotoxic structures that have the potential to be promising lead compounds for antineoplastic drugs.

Aciculatin is a natural compound isolated from the medicinal herb, *Chrysopogon aciculatus*, which is widely used for the treatment of swellings, common cold, fever, and diarrhea. Aciculatin was first reported in 1991 showing DNA-binding activity and cytotoxicity *in vitro* [1], [2]. A recent study demonstrated that aciculatin exerts dual inhibitory effects on inducible nitric oxide synthase (iNOS) and cyclooxygenase-2 (COX-2) due to regulation of the NF-kappaB and c-Jun N-terminal kinase (JNK)/p38 pathways [3]. However, the exact mechanism by which aciculatin exerts its tumor inhibitory effect remains unclear.

Tumor suppressor p53 plays a crucial role in arousing response to cellular stresses such as hypoxia, DNA damage, and oncogene activation. In normal circumstances, p53 is rapidly degraded by the E3-ubiquitin ligase murine double minute 2 (MDM2); the half-life of p53 is thus short and its protein level is difficult to detect [4]. Once cells are exposed to the aforementioned stresses, p53 degradation is turned off. Accumulated p53 then forms tetramers and binds to specific DNA domains, leading to transcriptional regulation of genes. The most common effects are DNA repair, cell cycle arrest, senescence, and apoptosis. The downstream cellular responses of p53 could suppress uncontrolled growth, and if necessary, eliminate damaged cells [5]. This strategy has been used in current cancer therapies to induce p53-dependent apoptosis using, for example, DNA damage agents. However, the side effects of this treatment are severe due to genotoxicity. Moreover, even though nearly 50% of human cancers have wild-type p53, some up-regulated pathways block p53 function such as p14ARF deficiency and MDM2 amplification [6]. Therefore, agents that can induce or restore wild-type p53 function and correct the abnormal pathway with lower side effects are ideal candidates for novel anti-cancer drugs [7], [8].

MDM2 oncoprotein is an E3 ubiquitin ligase that controls the p53 level in cells. MDM2 is a negative regulator mediating the ubiquitin degradation of p53; MDM2 also inhibits the transcriptional activity of p53 and facilitates its nuclear export [9]. Overexpression of MDM2 which impairs p53 function has been found in many human tumors. Moreover, MDM2 interacts with various tumor suppressor proteins including Rb, p21, p19/14^ARF^, E2F1, p73 and MTBP [10], [11], [12], [13], [14].

In this study, we aimed to identify the anti-tumor mechanism of aciculatin with *in vitro* and *in vivo* experiments using HCT116 human colorectal cancer cells. The results provide evidence of the importance of p53 and its downstream targets in aciculatin-induced cell cycle arrest and caspase-dependent apoptosis. The effects of aciculatin on MDM2 reduction and related p53 accumulation are also described. Same results were also confirmed using A549, a p53 wild-type non-small cell lung cancer cell line. Collectively, these findings suggest that aciculatin is a promising natural product for anticancer therapy.

## Materials and Methods

### Materials

Aciculatin was extracted and purified from *Chrysopogon aciculatus* by Professor C.C. Chen, Department of Biotechnology, Hungkuang University, Taiwan, with purity over 98%. RPMI 1640, fetal bovine serum (FBS), penicillin, streptomycin, and all other tissue culture reagents were obtained from Life Technologies (Grand Island, NY, USA). Enhanced chemiluminescence detection kit was from Amersham. Trizol reagent was from Invitrogen (Carlsbad, CA, USA); random primer and M-MLV RT were purchased from Promega (Madison, WI. USA); pro-Taq was from Protech (Taipei, Taiwan). HRP polymer conjugate reagent SuperPictureTM was from ZYMED LABORATORIES INC. (San Francisco, CA, USA); Nuceolin antibody, propidium iodide (PI), 3-(4, 5-dimethylthiazol-2-yl)-2,5-diphenyltetrazolium bromide, sulforhodamine B, and all of the other chemical reagents were obtained from Sigma Chemical (St. Louis, MO, USA).

### Cell culture

The human colorectal cancer cell line HCT116 and human non-small cell lung cancer cell line A549 were purchased from American Type Culture Collection (ATCC; Manassas, VA, USA). p53-KO (p53 knockout) HCT116 cells were kindly provided by Dr. B. Vogelstein (Johns Hopkins). Both were cultured in RPMI 1640 with 10% heat-inactivated fetal bovine serum (v/v) and penicillin (100 units/mL)/streptomycin (100 µg/mL). Cells were maintained in a humidified incubator at 37°C in 5% CO_2_/95% air.

### MTT assay

Cells were incubated with vehicle (0.1% DMSO) or compounds for 48 h. Washed once and incubated with 0.5 mg/mL 3-(4,5-dimethylthiazol-2-yl)-2,5-diphenyltetrazolium bromide at 37°C for 1 h. After the incubation, cells were lysed with DMSO and the absorbance was obtained using an ELISA reader (550 nm). Results were calculated as: cell viability (%) = average O.D. of wells/average O.D. of control wells.

### FACScan flow cytometry

After the treatment of vehicle (0.1% DMSO) or compounds for the indicated time courses, cells were harvested by trypsinization, for cell cycle analysis, cells were fixed with 70% (v/v) alcohol at 4°C overnight. After centrifugation, cells were incubated in 0.1 mol/L phosphate-citric acid buffer [0.2 mol/L NaHPO_4_, 0.1 mol/L citric acid (pH 7.8)] for 20 min at room temperature. Then, cells were centrifuged and resuspended with 0.5 mL PI solution containing Triton X-100 (0.1%, v/v), RNase (100 µg/mL), and PI (80 µg/mL). DNA content was analyzed with the FACScan and CellQuest software (Becton Dickinson). For annexin V-FITC and PI double staining, the FITC Annexin V Apoptosis Detection Kit (BD Biosciences; San Jose, CA, USA) was performed. Cells were centrifuged and incubated with these reagents immediately. After incubation for 15 min, the fluorescently labeled cells were then analyzed with the FACScan and CellQuest software.

### TUNEL

Cells seeded in 8-well slide chambers were starved overnight and treated with vehicle (0.1% DMSO) or aciculatin for 24 h. Cells and the tumor tissue slices were fixed in 4% paraformaldehyde and washed twice with PBS. The apoptotic cells were identified *in situ* using terminal deoxynucleotidyl transferase to transfer biotin-deoxyuridine triphosphatase (TUNEL, Roch Diagnostics, Mannheim, Germany) to the free 3′-OH of cleaved DNA. Cleavage sites were labeled by Biotin and visualized by reaction with fluorescein conjugated avidin (avidin-fluorescein isothiocyanate). Photomicrographs were obtained by Leica TCS SP2 confocal spectral microscope.

### Western blot analysis

Western blotting for total cell lysate and nuclear extraction were done as previously reported [15], [16] with anti- p53, anti-pRb and anti-caspase-8 (BD Biosciences; San Jose, CA, USA); anti-α-tubulin, anti-actin, anti-p21, anti-p27, anti-poly(ADP-ribose) polymerase, anti-phosphorylated H2AX (Ser139), anti-MDM2, anti-PUMA, HRP-conjugated anti-mouse, and anti-rabbit IgG (Santa Cruz Biotechnology; Santa Cruz, CA, USA); anti-caspase-3(Imgenex; San Diego, CA, USA); anti-caspase-9 and phospho-ser15-p53 (Cell Signaling Technology; Beverly, MA, USA).

### RNA extraction and reverse transcription-polymerase chain reaction(RT-PCR)

Total RNA was extracted with Trizol reagent by the manufacturer's protocol (Invitrogen, Carlsbad, CA, USA). Reverse transcription was performed with 5 µg mRNA and random primer at 65°C for 5 min, then mixed with Moloney murine leukemia virus reverse transcriptase (RT) to react at 37°C for 1 h to obtain cDNA. Gene amplification was followed with RT-polymerase chain reaction (PCR). The primer sequences used for amplification were as follows: GAPDH, 5′-TCCTTGGAGGCCATGTGGGCCAT-3′/5′-TGATGACATCAAGAAGGTGGTGAAG-3′; p53, 5′-CAGCCAAGTCTGTGACTTGCACGTAC-3′/5′-CTATGTCGAAAAGTGTTTCTGTCATC-3′; MDM2, 5′-GGGAGATATGTTGTGAAAGAAGC3′-/5′-CCCTGCCTGATACACAGTAACTT. Standard amplification parameters were used with the following processes: For p53, 94°C for 3 min, 35 cycles with denaturation at 94°C for 30 s, annealing at 60°C for 45 s, and extension at 72°C for 1 min, at last, 72°C for 10 min. For GAPDH and MDM2, 95°C for 10 min; 35 cycles (MDM2: 40 cycles) with denaturation at 95°C for 15 s, annealing at 60°C for 5 s, and extension at 72°C for 12 s; followed by a final extension at 75°C for 15 s. PCR products were analyzed on 1.5% agarose gel in the presence of 1 µg/mL of ethidium bromide.

### Transient transfection

HCT116 cells were seeded in 3.5-cm dishes overnight, and then transfected with TP53 siRNA or MDM2 plasmid using Lipofectamine 2000 according to the manufacturer's protocol. TP53 siRNA and lipofectamine 2000 are from Invitrogen (Carlsbad, CA, USA) and MDM2 plasmid (No. 16233) is from Addgene (Cambridge, MA, USA). After 6 h transfection and re-serum, cells were starved and then treated with vehicle (0.1% DMSO) or aciculatin for the indicated times. Cell lysates were collected for Western blot analysis.

### Immunohistochemistry analysis

The formalin-fixed paraffin-embedded (FFPE) tumor tissue slices were deparaffinized in xylene and rehydrated in a graded series of ethanol. Submerge tissue slices with 3% hydrogen peroxide to quench endogenous peroxidase activity. To unmask antigen, tissue slices were heated in 95°C Tris-EDTA Buffer (10 mM Tris Base, 1 mM EDTA Solution, 0.05% Tween 20, pH 9.0) for 30 min. Slices were blocked with 4% nonfat milk for 40 min and incubated with indicated antibody for 1 h at room temperature. HRP polymer conjugate reagent A (SuperPictureTM) were applied to the slices and reagent B for subsequently peroxidase catalyzation which visualizes the location of the antigen. Mayer's Hematoxylin solution was for counterstaining. The results were captured by Zeiss Axioskop-2 microscope.

### Tumor xenograft models

Male severe combined immunodeficient (SCID) mice were implanted s.c. with HCT116 tumor cells. When the tumors reached the average volume of 90 mm^3^, the mice were divided into two groups (*n* = 5) and the agent treatment was initiated. Vehicle (Cremophor EL/ethanol, 1∶1; 0.2 mL/mouse) or aciculatin (30 mg/kg) were administered intraperitoneally (i.p.) for five times each week. The length (*L*) and width (*W*) of the tumor were measured every 3 to 4 days, and the tumor volume was calculated as *LW* ^2^/2. The protocols of the *in vivo* study were approved by the Animal Care and Use Committee at National Taiwan University.

### Statistical analysis

Data were expressed as mean ± SEM of the indicated number for separate experiments. Statistical analysis of data was performed with one-way ANOVA followed by the *t* test, and *p*<0.05 were considered significant.

## Results

### Aciculatin induces G0/G1 arrest and subsequent apoptotic cell death in human cancer cells

**Figure 1:**
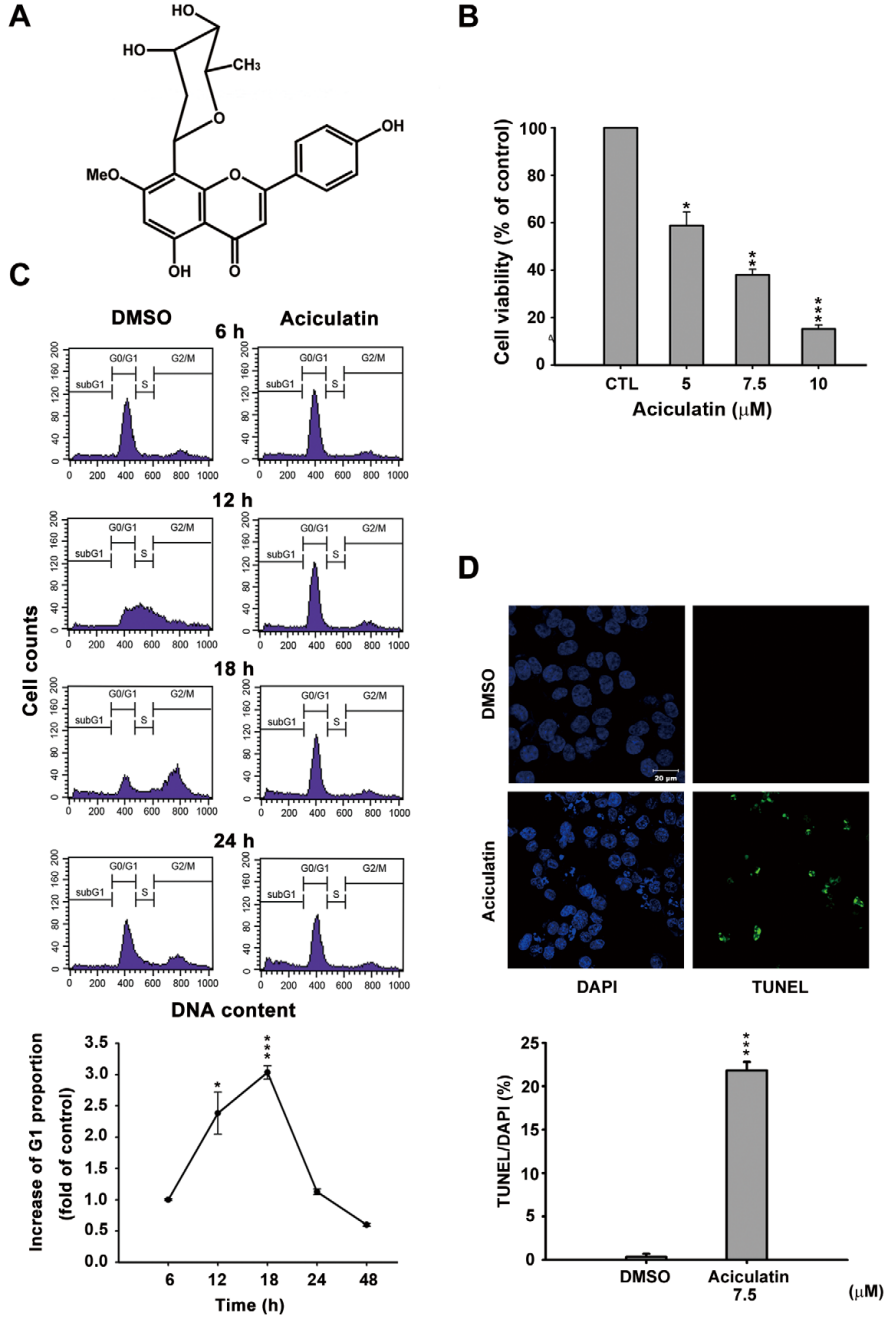
Effect of aciculatin on cell viability and cell cycle in human cancer cells. A, Structure of aciculatin: 8-(2,6-dideoxy-*β*-*ribo*-hexopyranosyl)-5-hydroxy- 2-(4-hydroxyphenyl)-7-methoxy-4H-1-benzopyran-4-one sesquihydrate. B, HCT116 cells were incubated with the indicated concentrations of aciculatin (5–10 µM) for 48 h. Cell viability was then determined by MTT assay. C, HCT116 cells were starved overnight, and then incubated with vehicle (0.1% DMSO) or aciculatin (10 µM) for the indicated period. The DNA content was subsequently analyzed by PI staining using a FACScan flow cytometric assay. The curve chart shows that aciculatin increased the G1 population of cells in a time-dependent manner. Mean ± SE values from 3 independent experiments. *P<0.05 and ***P<0.001, compared with non-treated cells. D, HCT116 cells were treated with vehicle (0.1% DMSO) or aciculatin (7.5 µM) and then double stained with TUNEL and DAPI. Increased green fluorescence indicated that the cells underwent apoptosis after aciculatin treatment (TUNEL, right panel). The nuclei were stained with DAPI (left panel). The bar chart shows the proportions of TUNEL positive cells in each treatment normalized to DAPI. ***P<0.001.

Aciculatin is a novel C-glycoside flavonoid derived from *C. aciculatus* extract (Figure 1A).The tight carbon-carbon bond makes a C-glycoside more stable than an O-glycoside in the acidic environment or the presence of glycosidases *in vitro* and *in vivo*. We used a sulforhodamine B assay (SRB) to demonstrate that aciculatin caused growth inhibition, not only in the HCT116 human colorectal cancer cell line (GI_50_ = 2.81 µM) but also in other cancer cell lines (Table S1). The cytotoxicity of aciculatin in HCT116 cells was determined by mitochondrial 3-(4,5-dimethylthiazol-2-yl)-2,5-diphenyltetrazolium (MTT) bromide reduction assay with an IC_50_ of 5.88 µM (Figure 1B). To determine the cell cycle distribution, synchronized cells were treated with aciculatin and analyzed with FACScan flow cytometry and propidium iodide (PI) staining. The results showed that aciculatin can induce G0/G1 arrest and subsequent accumulation of sub-G1 phase cells in a time-dependent manner. The increased G1 proportions after treatment at the indicated time points are shown in the curve chart (Figure 1C). We next investigated the induction of apoptosis by aciculatin using TUNEL assay. Figure 1D shows that DNA fragmentation increased after 24 h of 7.5 µM aciculatin treatment. The percentages of TUNEL positive cells in this assay were calculated and quantification data was shown. These data indicate that aciculatin can significantly induce apoptosis in HCT116 cells.

### Aciculatin induces cell cycle arrest and apoptosis through up-regulation of p21^WAF1/CIP1^ and induction of caspase activity

**Figure 2:**
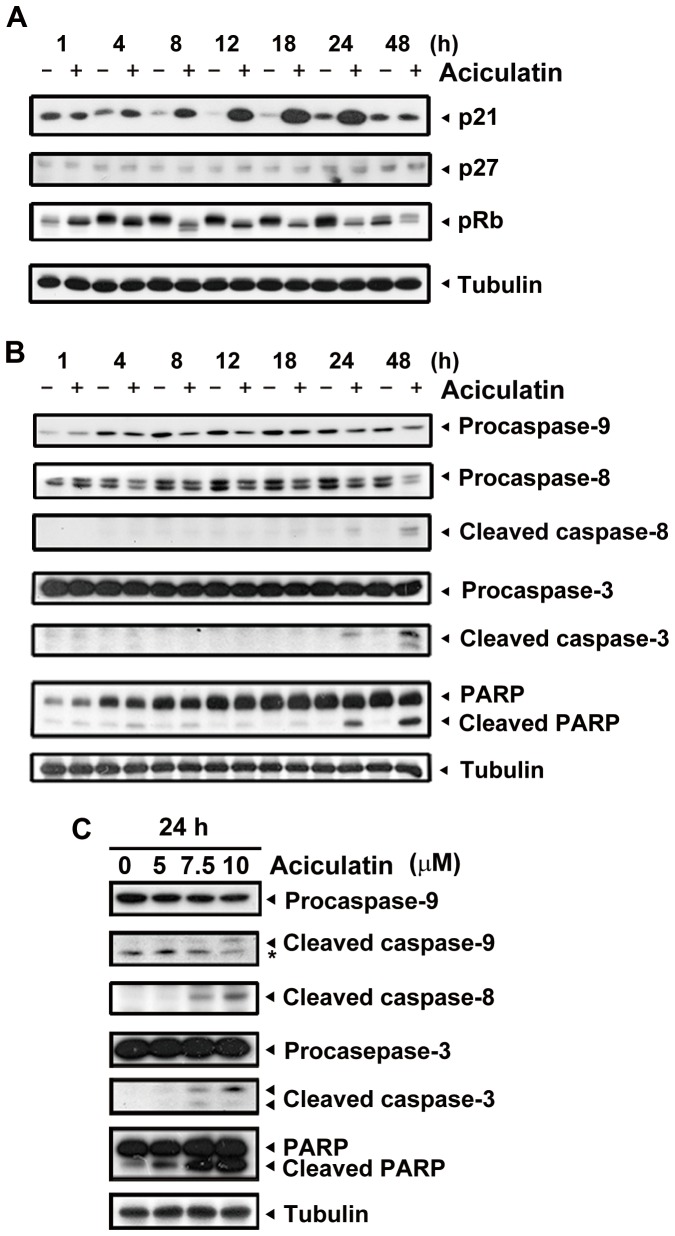
Effect of aciculatin on G0/G1-related proteins and apoptotic factors in HCT116 cells. A, HCT116 cells were treated with aciculatin (10 µM) for the indicated periods and then harvested for detection of G0/G1 arrest-related proteins (p21, pRb, and p27) by immunoblotting. B, HCT116 cells were treated with aciculatin (10 µM) for the indicated periods and then harvested for detection of caspase activation and PARP cleavage. C, HCT116 cells were treated with various concentrations of aciculatin (5, 7.5 and 10 µM) for 24 h. After aciculatin treatment, apoptotic proteins were detected for each treatment concentrations. The star marks a non-specific band.

We next examined the effect of aciculatin on G0/G1 arrest cell cycle regulatory proteins. The cyclin-dependent kinase inhibitors p21 and p27 are crucial regulators of G0/G1 arrest. As shown in Figure 2A, p21 was significantly upregulated and retinoblastoma protein (Rb) was dephosphorylated without any apparent change in p27 after 4 h of aciculatin treatment. Both intrinsic (caspase-9) and extrinsic (caspase-8) apoptotic pathways were detected after 8 h of treatment, and the downstream apoptotic effector caspase-3 was also activated (Figure 2B). Concentration-dependent increase in apoptosis was also identified 24 h after aciculatin treatment (Figure 2C). These results demonstrate that aciculatin treatment can trigger HCT116 cell cycle arrest at the G0/G1 phase and subsequently induce apoptosis.

### Aciculatin increased the expression of the p53 protein and its downstream effectors via a proteasome-mediated degradation pathway

**Figure 3:**
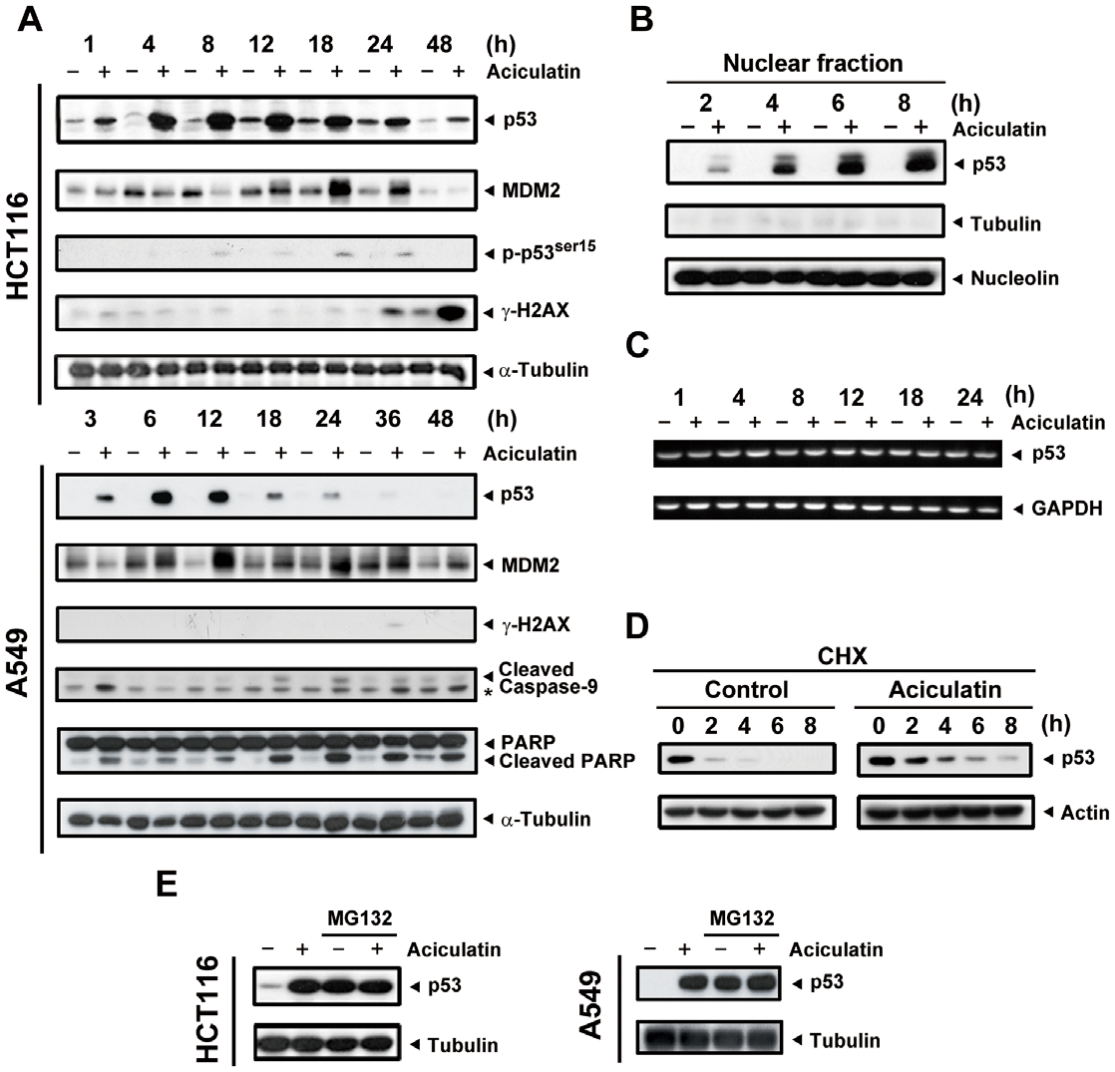
Accumulation of p53 is observed following aciculatin treatment in HCT116 and A549 cells via a proteasome degradation pathway. A, HCT116 and A549 cells were treated with aciculatin (10 µM) for the indicated periods and then harvested for detection of p53-related proteins. The levels of p53, phospho-ser15-p53, γ-H2AX, and the p53 downstream target MDM2 were then determined in HCT116 cells. The levels of p53, MDM2, γ-H2AX, cleaved caspase-9 and PARP were determined in A549 cells. The star marks a non-specific band. B, Nuclear extraction of HCT116 cells was performed after aciculatin treatment at the indicated time points. Aciculatin-induced nuclear accumulation of p53 was shown to be time-dependent. C, HCT116 cells were treated with aciculatin (10 µM) at different time points followed by extraction of total RNA. The p53 mRNA was co-amplified with GAPDH. D, HCT116 cells were pretreated with aciculatin (10 µM) for 3 h, followed by treatment with cycloheximide (20 µg/ml) with or without aciculatin (10 µM) for the indicated periods. E, HCT116 and A549 cells were co-treated with 10 µM MG132 and 10 µM aciculatin for 6 h and then harvested for p53 detection by immunoblotting.

The phenomena our prior data revealed are believed to be associated with the tumor suppressor protein p53. Thus, the expression of p53 was examined at a series of time points. As shown in Figure 3A, aciculatin significantly induced p53 expression in a time-dependent manner. The p53 downstream product MDM2, which is also a major regulator of ubiquitin-mediated p53 degradation, was found to be upregulated following the accumulation of p53. Interestingly, there was a decrease in MDM2 during the first few hours of aciculatin treatment. We then confirmed the correlation between aciculatin and DNA damage. Our data indicate that aciculatin only slightly increased phospho-ser15-p53, which is the downstream effect of DNA damage. The DNA double-strand break marker phosphorylated histone 2AX (γ-H2AX) appeared until the later stages of aciculatin treatment. In addition, to check DNA single and double strand breaks, single-cell gel electrophoresis assay (COMET assay) was performed. The results showed that there is no obvious DNA damage after 6 h treatment of 10 µM aciculatin with a novel DNA damage agent QS-ZYX-1-61 (published by our group previously) as the positive control [17]. (Figure S1). These results indicate that DNA damage is not an obvious early effect of aciculatin treatment. We than verified this p53-dependent effect in another p53 wild type cancer cells A549. Aciculatin also triggered p53 accumulation, early MDM2 depletion (3 h) and the subsequent apoptosis pathway including cleaved caspase-9 and cleaved PARP in A549. To investigate the protein level of transcription factor p53 accumulated in nucleus by aciculatin, the nuclear fractions were extracted and analyzed. Figure 3B shows that p53 was increased in nucleus in a time-dependent manner after aciculatin treatment. To determine the mechanism of aciculatin-induced p53 accumulation, we first determined the mRNA level of p53. Figure 3C shows that there was no prominent change in p53 mRNA level. In normal circumstances, p53 protein has a very short half-life (approximately 15–30 min) due to rapid proteasome degradation. Therefore, we determined whether aciculatin increases half-life of p53. When cycloheximide was used to inhibit protein biosynthesis, we observed that aciculatin-induced p53 was stabilized and had a longer half-life (Figure 3D). Furthermore, we introduced the proteasome inhibitor MG132 to block the degradation pathway. Figure 3E shows that the protein level of p53 was not increased by aciculatin treatment after MG132 pre-treatment in HCT116 and A549 cells. These results demonstrated that the proteasome-mediated degradation pathway is important for aciculatin-induced p53 accumulation.

### Aciculatin decreases MDM2 at a transcriptional level, which is critical for p53 accumulation

**Figure 4:**
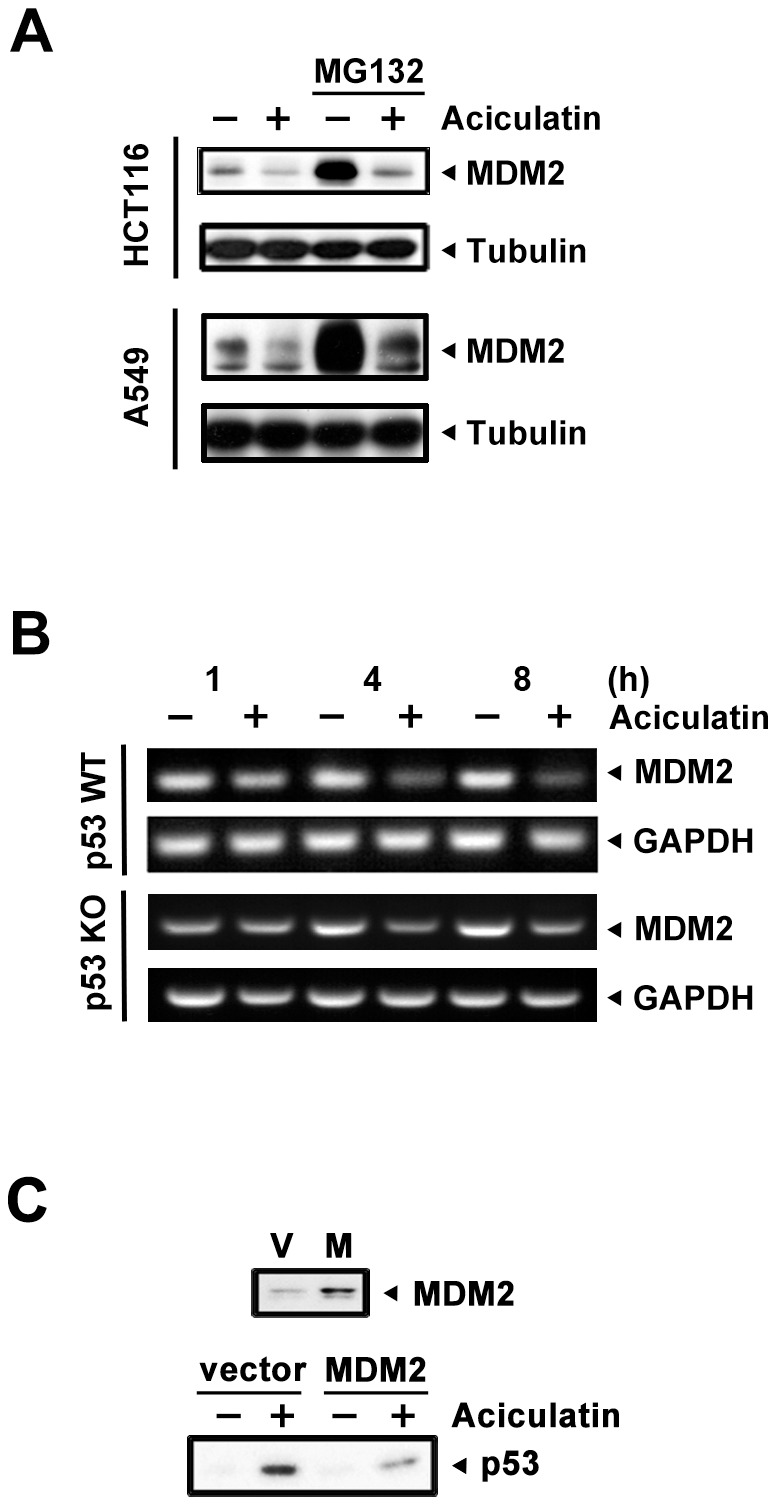
Aciculatin treatment attenuates MDM2 mRNA, contributing to p53 accumulation. A, HCT116 and A549 cells were co-treated with 10 µM MG132 and 10 µM aciculatin for 6 h (HCT116) and 3 h (A549) then harvested for detection of MDM2 by immunoblotting. B, HCT116 and p53-KO HCT116 cells were treated with aciculatin (10 µM) at different time points followed by extraction of total RNA. The MDM2 mRNA was co-amplified with GAPDH. C, The MDM2 plasmid was introduced into the HCT116 cells to induce over-expression of the MDM2 protein; the cells were then treated with aciculatin for 3 h. Protein levels of p53 and MDM2 were detected by western blotting.

In our previous study, no obvious DNA damage signals have been found. This may imply that this pathway is not the only mechanism contributing to p53 accumulation. MDM2 mediates p53 ubiquitination, leading to proteasomal degradation of p53. MDM2 also controls its own degradation by auto-ubiquitylation [18]. In this study, MDM2 depletion was found in the early stages of aciculatin treatment (Figure 3A). Thus, we next evaluated the mechanism of MDM2 ablation to determine whether this decrement was involved in aciculatin-induced p53 accumulation. After co-treatment with aciculatin and MG132, a proteasome inhibitor, the MDM2 protein level still declined remarkably in HCT116 and A549 cells (Figure 4A). These results indicate that other proteasome-independent regulatory pathways are active. We then examined the mRNA level and found that MDM2 mRNA started to decrease after 1 h of aciculatin treatment. Same result can be achieved in p53-KO HCT116, suggesting that aciculatin induced MDM2 depletion is not a p53-related effect (Figure 4B). To identify the importance of MDM2 in p53 accumulation, we investigated aciculatin-induced p53 accumulation after over-expressing MDM2 in HCT116 cells. Following aciculatin treatment, over-expression of MDM2 reversed the p53 level (Figure 4C). Collectively, these results indicate that aciculatin can induce accumulation of p53 through MDM2 mRNA depletion.

### Role of p53 in aciculatin-mediated cell cycle arrest and apoptosis

**Figure 5:**
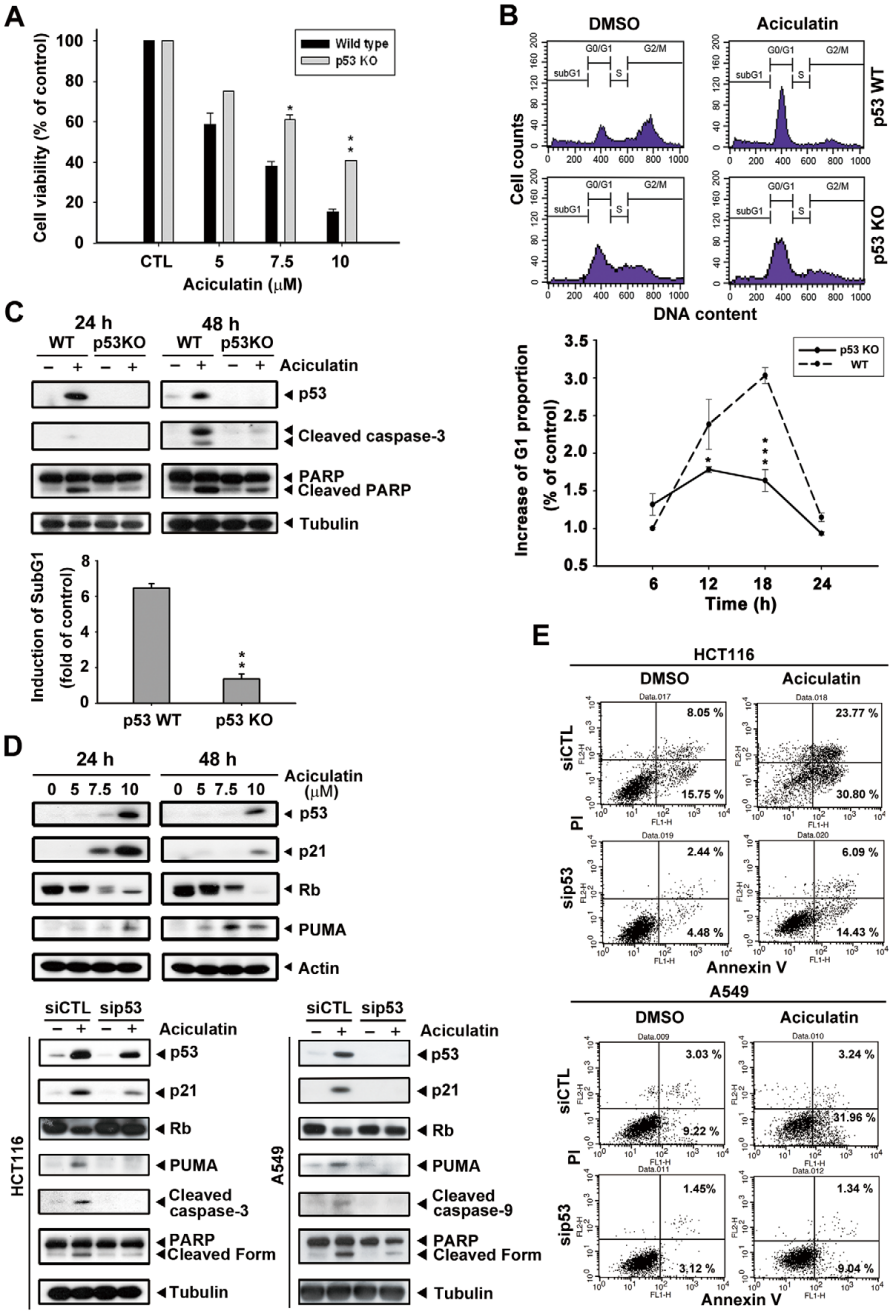
p53 is required for aciculatin to trigger cell cycle arrest and apoptosis. A, p53-KO HCT116 cells were incubated with aciculatin (5–10 µM) for 48 h. Cell viability was then determined by MTT assay. The results were compared with wild-type HCT116 cells. Mean ± SE from 3 independent experiments. *P<0.05 and **P<0.01. B, p53-KO HCT116 cells were starved overnight and then incubated with vehicle (0.1% DMSO) or aciculatin (10 µM) for different periods. The DNA proportion was subsequently analyzed by PI staining. The cell cycles of p53-WT and p53-KO HCT116 cells after treatment of 18 h are shown. The G1 proportions of both cell lines were compared. Mean ± SE values from 3 independent experiments. *P<0.05 and ***P<0.001. C, p53-WT and p53-KO HCT116 cells were incubated with aciculatin (10 µM) for 24 h and 48 h and then harvested for detection of p53, caspase activation, and cleavage of PARP (upper). p53-WT and p53-KO cells were treated with vehicle (0.1% DMSO) or aciculatin (10 µM) for 24 h and were subsequently analyzed by PI staining. The induction of sub-G1 phase of each group was determined (lower bar chart). **P<0.005. D, HCT116 cells were treated with various concentrations of aciculatin (5, 7.5, and 10 µM). After 24 h and 48 h aciculatin treatment, The levels of p53, p21, pRb and PUMA were then determined (upper). p53 siRNA was used to knock down the p53 level of HCT116 and A549 cells, which were then treated with aciculatin for 24 h. The cells were harvested for detection of p53 and apoptotic related proteins (lower). E, p53 knock-down HCT116 and A549 cells were treated with aciculatin (10 µM) for 40 h and then double stained with annexin V-FITC and PI. The percentages of fluorescently labeled cells were determined by flow cytometry.

Next, we aimed to identify the role of p53 in aciculatin-induced HCT116 cell cycle arrest and apoptosis. First, we compared the cytotoxicity of aciculatin in a HCT116 p53-WT (wild-type) and p53-KO (knockout) system. As shown in Figure 5A, we found a significant difference in the IC_50_ between these 2 cell lines; the WT cells (IC_50_ value, 5.88 µM) were more sensitive to aciculatin than the p53-KO cells (IC_50_ value, 9.13 µM). Furthermore, the proportion of aciculatin-induced G0/G1 arrest in the WT cells was about 3-fold higher than that in the p53-KO cells after 18 h of aciculatin treatment (Figure 5B). We then used western blotting to examine p53 and apoptotic proteins in p53-WT and p53-KO HCT116 cells during aciculatin treatment. Figure 5C shows that the p53-KO cells expressed lower levels of caspase-3 and cleaved poly (ADP-ribose) polymerase (PARP). The induction of sub-G1 was also examined after aciculatin treatment and results indicate that p53-KO cells were more resistant to aciculatin-induced sub-G1 increment (lower bar chart). The p53 direct transcriptional targets, p21 and PUMA (p53 upregulated modulator of apoptosis) are related to growth arrest and apoptosis. Both of them were upregulated after 24 h and 48 h of aciculatin treatment in a concentration-dependent manner. (Figure 5D). Figure 5D also shows knockdown of the p53 protein level by siRNA in p53 wild-type cells HCT116 and A549; this resulted in significant inhibition of p53 expression after aciculatin treatment, an effect that lasted for up to 24 h. This p53 depletion led to abrogation of aciculatin-induced p21 and dephosphorylation of Rb; also the apoptotic proteins PUMA, caspase-9, caspase-3 and PARP. After cells were double stained with annexin V-FITC and PI for apoptosis and necrosis, the results revealed that p53 knock-down HCT116 and A549 cells were both more resistant to aciculatin induced apoptosis than wild-type cells (Figure 5E). Based on these data, we suggest that p53 is a pivotal mediator of aciculatin-induced apoptosis.

### Aciculatin inhibited HCT116 tumor cell growth in the mouse xenograft models

**Figure 6:**
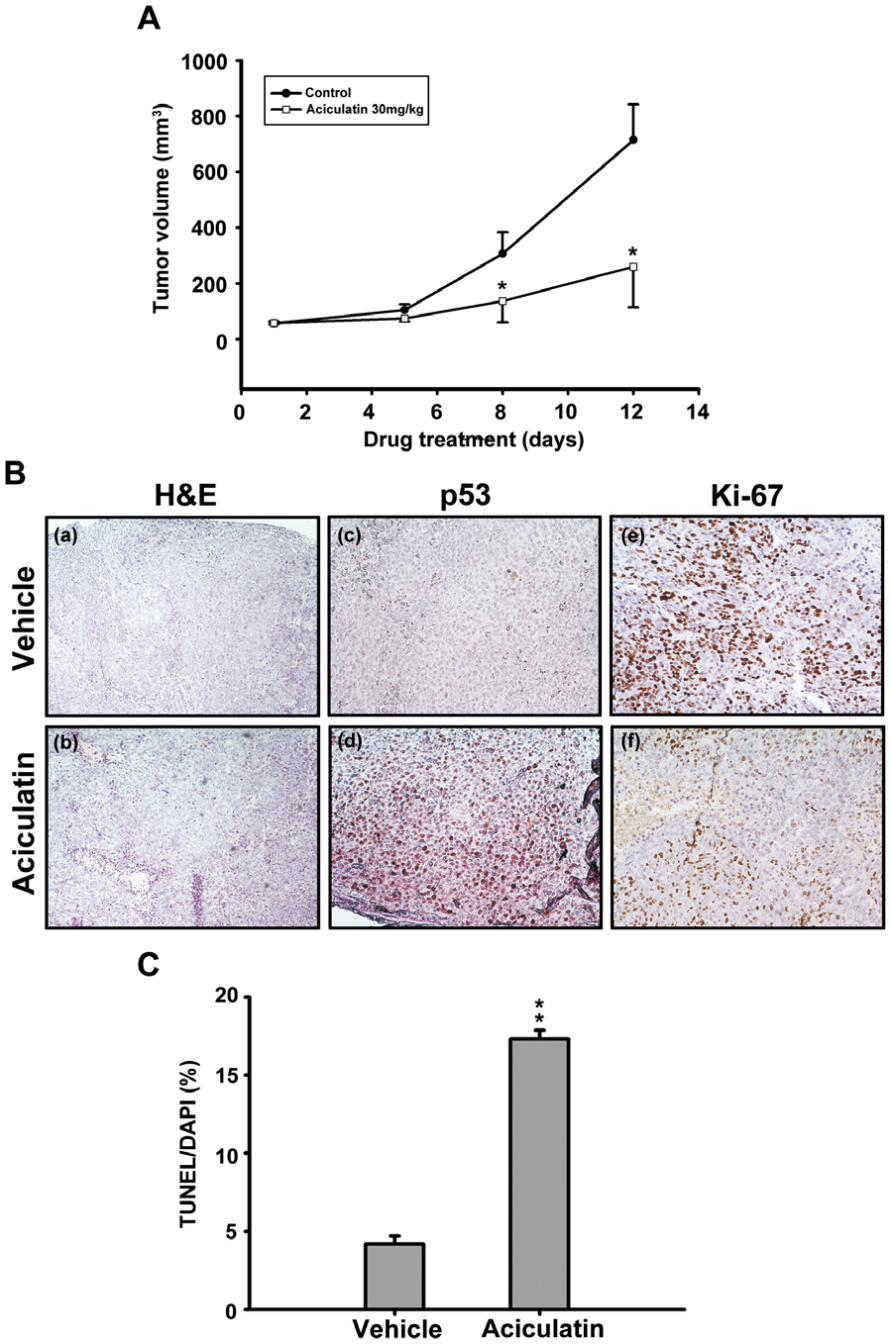
Aciculatin anti-cancer activity in the HCT116 xenograft models. HCT116 cells were injected subcutaneously into the flanks of severe combined immunodeficient mice. The mice were divided into two groups (*n* = 5) and when the tumors reached the average volume (90 mm^3^) the treatment was initiated. The mice were sacrificed on day 12 thereafter. A, The curves show the mean (±SE) tumor sizes measured within each group. The differences in tumor size between the control and treated mice were statistically significant (*P<0.05). Bodyweight was measured every day from day 1 of administration. There were no significant differences after treatment in any of the groups (data not shown). B, Treated and untreated tumor slices were assessed by H&E (a, b) and IHC staining for p53 protein (c, d) and Ki-67 protein (e, f). Tumor samples were observed under 100× (for H&E) and 200× (for IHC) magnification. C, The tumor tissue slices were assessed by TUNEL assay kit and the bar charts indicates the fold change of sub-G1 phase. **P<0.01.

To examine the *in vivo* anti-cancer activity of aciculatin, we established HCT116-derived xenograft models in severe combined immunodeficient (SCID) mice. Both control and treated mice were sacrificed, and the HCT116-xenografted tumor tissues were examined. As shown in Figure 6A, our results demonstrated that intraperitoneal administration of aciculatin (30 mg/kg) once daily significantly inhibited tumor growth from day 8, without any loss of body weight; this suggests that there was no obvious cytotoxicity *in vivo*. We also stained the HCT116-xenografted tumor tissues with Hematoxylin and Eosin (Figure 6B–a, b), p53 antibody (Figure 6B–c, d) and Ki-67 (Figure 6B–e, f). The results revealed that treatment with aciculatin significantly increased p53 expression and the cell proliferation marker Ki67. These observation correlates with the *in vitro* studies. The tumor tissue slices were also stained with TUNEL reagent and the results indicates that aciculatin-treated tumor did undergo apoptosis (Figure 6C).

## Discussion

The tumor suppressor protein p53 has been a promising target of anti-cancer therapy for decades. It plays an essential role in cell cycle regulation and apoptosis. Here, we demonstrated that aciculatin is a compelling p53 inducer and also activates the downstream effectors of p53. p21, a well-known transcriptional target of p53, is the major regulator of the G0/G1 cell cycle phase. As shown in Figure 2A and 5D, p21 expression was increased and pRb was hypophosphorylated after aciculatin treatment. p21 has previously been shown to bind to Cdk/cyclin complexes, inducing hypophosphorylated pRb to sequestrate E2 gene promoter region binding factor 1 (E2F-1), thereby inhibiting the ability of E2F-1 to carry out the cell cycle [19]. Some studies have indicated that HCT116 cells are prone to remain in G0/G1 phase rather than undergo apoptosis with p53 induction [20], [21]. In this situation, p21 induction is regarded as protection and mediates cell cycle arrest to suppress apoptosis [5]. However, a high percentage of HCT116 cells do undergo apoptosis after G0/G1 arrest, as was shown here with the aciculatin-treated cells. Furthermore, a recent study suggested that p21 induced by nongenotoxic p53 activation, unlike stress-induced p53 activation, does not affect the apoptotic response [22], which could explain the role of p21 induced by nongenotoxic aciculatin. Another p53 downstream target, PUMA, was also upregulated after aciculatin treatment (Figure 5D). PUMA is a potent pro-apoptotic factor and has been reported to mediate p53-dependent apoptosis [20], [23]. These results indicate that the aciculatin-induced p53 pathway is growth inhibitory and cytotoxic in HCT116 cells.

Aciculatin is a C-glycosidic flavonoid with more indestructible structure than O-glycosidic flavonoids. Several flavonoids such as wogonin, apigenin, luteolin, and quercetin have been documented to induce p53 activity [23], [24]. Here, aciculatin induced accumulation of p53 by inhibiting proteasome-dependent degradation at lower concentrations compared to other flavonoids (Figure 3C–E). It has been reported that p53 degradation is decreased in response to cellular stresses, especially DNA damage [8]. Recently, flavonoid interactions with DNA and RNA have been studied [25], and aciculatin has been documented to have DNA-binding ability [2]. Therefore, we checked for DNA damage after aciculatin treatment of HCT116 cells. However, no significant DNA damage was observed in the aciculatin-treated HCT116 cells. The histone H2AX is immediately phosphorylated to γ-H2AX when there is a DNA double-strand break in chromatin [26]. In this study, γ-H2AX did not appear until 24 h after aciculatin treatment, and this delayed effect was more likely a consequence of apoptosis. The COMET assay also proved no significant DNA breaks in early stage of aciculatin treatment. But our western blotting results still revealed faint upregulation of phospho-ser15-p53, which correlates with DNA damage and may contribute to the transcriptional activity of p53. Collectively, these results suggest that aciculatin is a DNA-binding agent, but one that does not induce significant DNA damage. Mechanisms other than genomic damage could be responsible for the p53 upregulation and anti-cancer activity of aciculatin.

Therapeutic agents that can activate the p53 pathway without genotoxicity are potentially safer agents to use in cancer therapy [7], [27]. This concept has already been considered in the development of drugs targeting the p53-MDM2 interaction. In this study, the ability of aciculatin to ablate MDM2 mRNA was noted (Figure 4B). Although the abrogation of MDM2 mRNA by some natural products, including genistein and gambogic acid, has been reported [28], [29], to our knowledge this is the first time to prove that overexpressed MDM2 could partially reverse the flavonoid-induced p53 accumulation in HCT116 cells. Phosphorylation of p53 (Figure 3A) could interrupt the p53-MDM2 interaction; this may resulted in the residual p53 level when MDM2 over-expression (Figure 4C). The contradictory observation that p53 stability is not responsive to changes in endogenous MDM2 level has also been reported [30]. However, we demonstrated that the depletion of MDM2 dose plays an important role in aciculatin-induced p53 stabilization in HCT116 cells.

We also found that aciculatin can still trigger cell death in p53-KO HCT116 and HT-29 (with mutant p53) (Table S1) cells via a p53-independent mechanism. Moreover, we proved that aciculatin could also deplete the MDM2 mRNA level of p53-KO HCT116 cells (Fig. 4B), which may contribute to the p53-independent cell death. Several studies have indicated that MDM2 interacts not only with p53 but also with other pivotal proteins such as p73 and p21 in tumor cells without wild-type p53 [31], [32], [33]. Thus, these MDM2 related effects may contribute to p53-independent cell death.

As demonstrated in our *in vivo* xenograft models, aciculatin effectively inhibited HCT116 tumor progression with no obvious toxicity (Figure 6A). This finding supports the contention that aciculatin does exhibit anti-tumor activity in living organisms. Immunohistochemical (IHC) staining showed that the p53 protein was upregulated in aciculatin-treated tumor tissues. This proved the importance of p53 *in vivo* (Figure 6B).

In conclusion, these results show that aciculatin is a potent p53 inducer and potent anti-tumor flavonoid with low genotoxicity. This study also provides insights into the relationship between MDM2 depletion and p53 accumulation. An interesting question that remains to be answered is whether there is any correlation between these promising effects and DNA-binding activity. If so, the integrity of the aciculatin structure becomes important, and the uncommon tight C-glycoside linkage could conserve the compound, rendering it indestructible in the cellular environment. We believe that aciculatin is a promising anti-cancer agent and that further aciculatin-related effects should be elucidated in future.

## Supporting Information

Figure S1 HCT116 cells were treated with 10 µM aciculatin for 6 h or 3 µM QS-ZYX-1-61 for 1 h as a positive control. After treatments, the single cell gel electrophoresis assay (comet assay) was performed to detect the DNA strand breaks. Damaged cell is indicated by arrow. The percentages of tailing cells were calculated in 3 different areas each group (**P<0.01).
(PDF)

Table S1 Various types of cancer cell lines were treated with aciculatin. Cell growth inhibitory activity (GI_50_) was determined by the SRB assay.
(PDF)
