# Supplementary material for: Aciculatin Induces p53-Dependent Apoptosis via MDM2 Depletion in Human Cancer Cells In Vitro and In Vivo
Source: PLoS One. 2012 Aug 13;7(8):e42192. doi: 10.1371/journal.pone.0042192 (PMC3418269; doi:10.1371/journal.pone.0042192)
Supplement: Figure S1 — HCT116 cells were treated with 10 µM aciculatin for 6 h or 3 µM QS-ZYX-1-61 for 1 h as a positive control. After treatments, the single cell gel electrophoresis assay (comet assay) was performed to detect the DNA strand breaks. Damaged cell is indicated by arrow. The percentages of tailing cells were calculated in 3 different areas each group (**P<0.01). (PDF) [file pone.0042192.s001.pdf]

Supplemental Fig.1

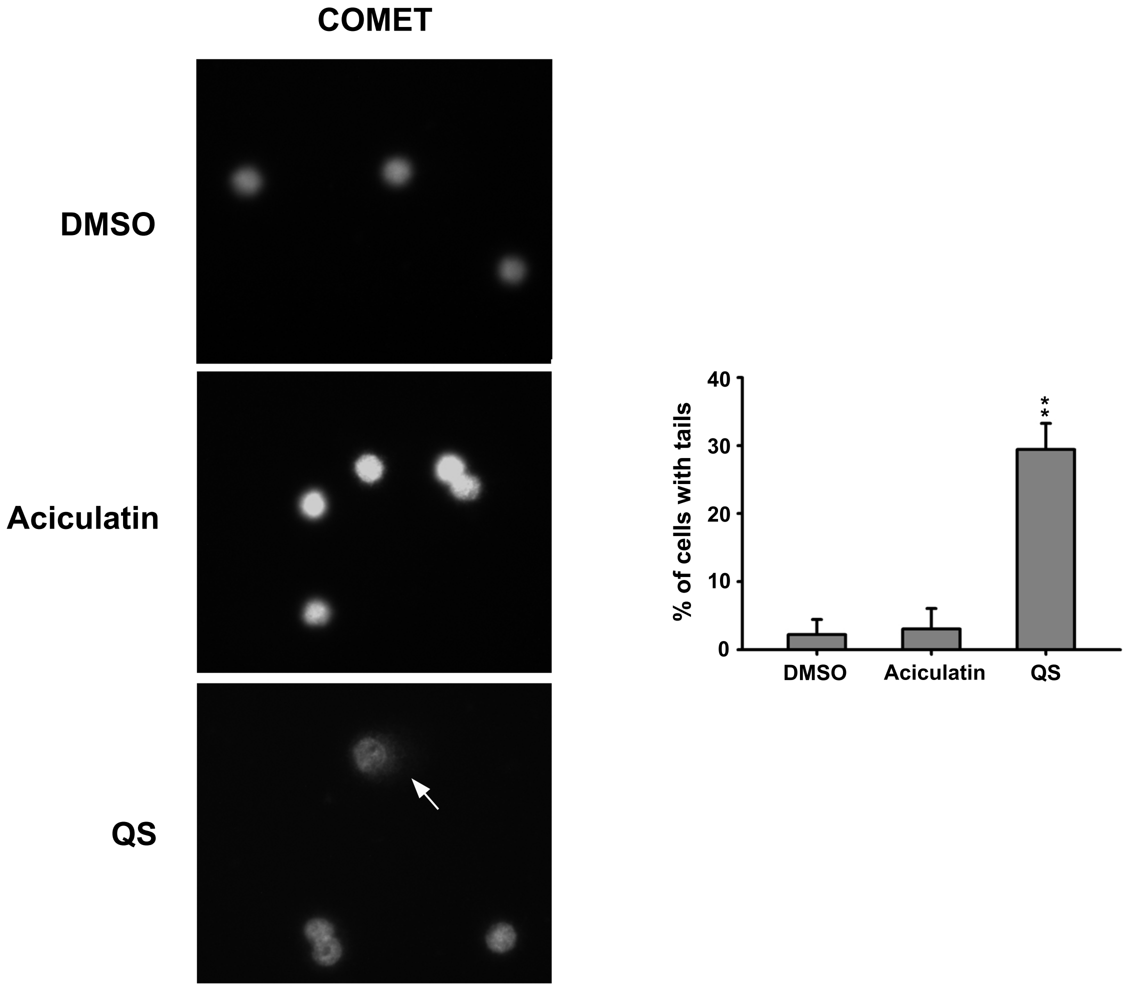

S.1 HCT116 cells were treated with 10 μM aciculatin for 6 h or 3 μM QS-ZYX-1-61 for 1 h as a positive control. After treatments, the single cell gel electrophoresis assay (comet assay) was performed to detect the DNA strand breaks. Damaged cell is indicated by arrow. The percentages of tailing cells were calculated in 3 different areas each group (*P* < 0.01).**
