# Supplementary material for: Aciculatin Induces p53-Dependent Apoptosis via MDM2 Depletion in Human Cancer Cells In Vitro and In Vivo
Source: PLoS One. 2012 Aug 13;7(8):e42192. doi: 10.1371/journal.pone.0042192 (PMC3418269; doi:10.1371/journal.pone.0042192)
Supplement: Table S1 — Various types of cancer cell lines were treated with aciculatin. Cell growth inhibitory activity (GI50) was determined by the SRB assay. (PDF) [file pone.0042192.s002.pdf]

Supplemental table 1

GI_{50} from SRB assay (μM)

HT-29	A549	PC-3	HCT116	Hep3B
10.2	7.2	2.8	2.8	2.5

Table 1: Various types of cancer cell lines were treated with aciculatin. Cell growth inhibitory activity (GI_{50}) was determined by the SRB assay.
